# Statin use is associated with the reduction in hepatocellular carcinoma recurrence after liver surgery

**DOI:** 10.1186/s12885-022-09192-1

**Published:** 2022-01-21

**Authors:** Elias Khajeh, Arash Dooghaie Moghadam, Pegah Eslami, Sadeq Ali-Hasan-Al-Saegh, Ali Ramouz, Saeed Shafiei, Omid Ghamarnejad, Sepehr Abbasi Dezfouli, Christian Rupp, Christoph Springfeld, Carlos Carvalho, Pascal Probst, Seyed Mostafa Mousavizadeh, Arianeb Mehrabi

**Affiliations:** 1 Division of Liver Surgery, Department of General, Visceral, and Transplantation Surgery, University of Heidelberg, Im Neuenheimer Feld 420, 69120 Heidelberg, Germany; 2 Department of Gastroenterology, University of Heidelberg, Heidelberg, Germany; 3 Liver Cancer Center Heidelberg (LCCH), Heidelberg, Germany; 4 Department of Medical Oncology, Heidelberg University Hospital, National Center for Tumor Diseases, Heidelberg, Germany; 5 Digestive Unit, Clinical Oncology, Champalimaud Clinical Centre, Lisboa, Portugal

**Keywords:** Hepatocellular carcinoma, Liver resection, Liver transplantation, Statins, Meta-analysis

## Abstract

**Background:**

Hepatocellular carcinoma (HCC) is the sixth most common form of cancer worldwide. Although surgical treatments have an acceptable cure rate, tumor recurrence is still a challenging issue. In this meta-analysis, we investigated whether statins prevent HCC recurrence following liver surgery.

**Methods:**

PubMed, Web of Science, EMBASE and Cochrane Central were searched. The Outcome of interest was the HCC recurrence after hepatic surgery. Pooled estimates were represented as hazard ratios (HRs) and odds ratios (ORs) using a random-effects model. Summary effect measures are presented together with their corresponding 95% confidence intervals (CI). The certainty of evidence was evaluated using the Grades of Research, Assessment, Development and Evaluation (GRADE) approach.

**Results:**

The literature search retrieved 1362 studies excluding duplicates. Nine retrospective studies including 44,219 patients (2243 in the statin group and 41,976 in the non-statin group) were included in the qualitative analysis. Patients who received statins had a lower rate of recurrence after liver surgery (HR: 0.53; 95% CI: 0.44–0.63; *p* < 0.001). Moreover, Statins decreased the recurrence 1 year after surgery (OR: 0.27; 95% CI: 0.16–0.47; *P* < 0.001), 3 years after surgery (OR: 0.22; 95% CI: 0.15–0.33; *P* < 0.001), and 5 years after surgery (OR: 0.28; 95% CI: 0.19–0.42; *P* < 0.001). The certainty of evidence for the outcomes was moderate.

**Conclusion:**

Statins increase the disease-free survival of patients with HCC after liver surgery. These drugs seem to have chemoprevention effects that decrease the probability of HCC recurrence after liver transplantation or liver resection.

## Background

Hepatocellular carcinoma (HCC) is the sixth most common malignancy [1] and is emerging as the fastest-growing fatal cancer in the United States with a rapidly rising mortality rate worldwide [2]. Establishing the best treatment option for HCC is difficult and depends on the tumor stage at the time of diagnosis [3]. Surgical or interventional curative approaches such as local ablation, surgical resection, and liver transplantation are the treatments of choice for tumors diagnosed in earlier stages [4–6]. However, detecting early-stage tumors is not feasible in many cases and only 13% of HCC cases are diagnosed early enough for curative therapy [7]. Although surgical procedures like tumor resection or liver transplantation have acceptable cure rates, they also have high recurrence rates [8, 9], with recurrence in more than 50% of patients after 5 years of surgery [10, 11]. Recurrence rates after transplantation are between 8 and 21% despite the use of new predictive models [12].

Both animal and human studies have shown an independent relationship between cholesterol levels and HCC progression [13]. HCC cell lines use cholesterol in their cell membranes and for organelle division [14] and levels of high-density lipoprotein cholesterol have been associated with tumor aggressiveness [13]. Statins are hydroxymethyl glutaryl coenzyme A (HMG-CoA) reductase inhibitors and are commonly used to lower cholesterol levels in blood [15]. In addition, statins have immunomodulatory effects and can protect against cancer [16–19]. Several studies have attempted to investigate the possible role of statins in preventing HCC recurrence [20]. Early work showed that statins affect molecular pathways in HCC cell lines to prevent over-proliferation in vivo [21]. These anti-cancer effects make statins an interesting candidate for HCC prevention [14].

Recent studies have suggested that statins might increase survival rates in HCC patients and reduce HCC recurrence rates after curative treatment [22, 23]. In this systematic review and meta-analysis, we investigate the role of statins in preventing HCC recurrence after hepatic surgery.

## Methods

The present study was reported according to the Preferred Reporting Items for Systematic Reviews and Meta-Analyses (PRISMA) guidelines and recommendations of the Study Center of the German Society of Surgery [24, 25].

### Eligibility criteria

The research question and eligibility criteria were formulated based on the PICOS strategy (population, intervention, comparison, outcomes, and design of studies).

- ▪ *Population:* All patients with HCC who underwent hepatic surgery, including liver resection and liver transplantation
- ▪ *Intervention:* Treatment with statins
- ▪ *Comparators:* No statin treatment
- ▪ *Outcome:* HCC recurrence after surgery
- ▪ *Study design:* Any study design except case reports, study protocols, animal studies, conference papers, and letters to the editor.

To eliminate the risk of analyzing the same patients more than once, the studies were thoroughly assessed and double publications and overlapping reports were excluded. The remaining studies were selected for full-text review by reviewing the titles and abstracts for eligibility.

### Literature search

The predefined search terms were: (“carcinoma, hepatocellular” OR “adenoma hepatocellular” OR “adenomas hepatocellular” OR “hepatocellular adenoma” OR “HCC” OR “hepatocellular carcinoma”) AND (“Statin” OR “Hydroxymethylglutaryl-CoA Reductase Inhibitors” OR “HMG-CoA Reductase Inhibitor”). Our comprehensive literature search was conducted in Medline/PubMed, EMBASE, Web of Science and Cochrane Central databases from their inception until February 2021. We also searched PubMed/Medline and Cochrane Central for systematic reviews of randomized clinical trials on surgical interventions. All studies comparing HCC recurrence in adult patients who underwent liver surgery were included.

### Study selection

Two authors (ADM and PE) independently screened all titles and abstracts and made their selections according to PICOS eligibility criteria. The full texts of appropriate studies were evaluated and their data were extracted by two authors (SAHS and AR) independently. Discrepancies were resolved through discussions with the first and senior authors (EK and AM). For each treatment group, the following data were extracted: study characteristics, patient characteristics, study quality, and outcome measures.

### Critical appraisal

The quality of each study was assessed by two independent reviewers (SAHA and AR) using the methodological index for non-randomized studies (MINORS). Quality was determined based on 12 MINORS items and was scored as follows: 0 (not reported), 1 (reported but inadequate) or 2 (reported and adequate). The best score was 24 for comparative studies. Studies with 20 points or lower were deemed high risk of bias, 21–23 points intermediate risk of bias, and 24 points low risk of bias. The overall quality of the evidence for each outcome was also assessed using Grading of Recommendations Assessment, Development and Evaluation (GRADE) approach.

### Statistical analysis

All data were analyzed by RevMan version 5.3 (Nordic Cochrane Centre, Cochrane Collaboration, Copenhagen, Denmark). The effect size for dichotomous outcomes were presented as odds ratios (OR) or hazard ratios (HR) with their corresponding 95% confidence intervals (CI). For analyzing ORs and HRs of included studies, random effects model was used. Statistical heterogeneity was evaluated with the I^2^ statistic. I^2^ values of 0–25% indicate insignificant heterogeneity, 26–50% indicate low heterogeneity, 51–75% indicate moderate heterogeneity, and 76–100% indicate high heterogeneity. A *p*-value less than 0.05 was considered statistically significant in all analyses.

## Results

### Literature search

**Fig. 1:**
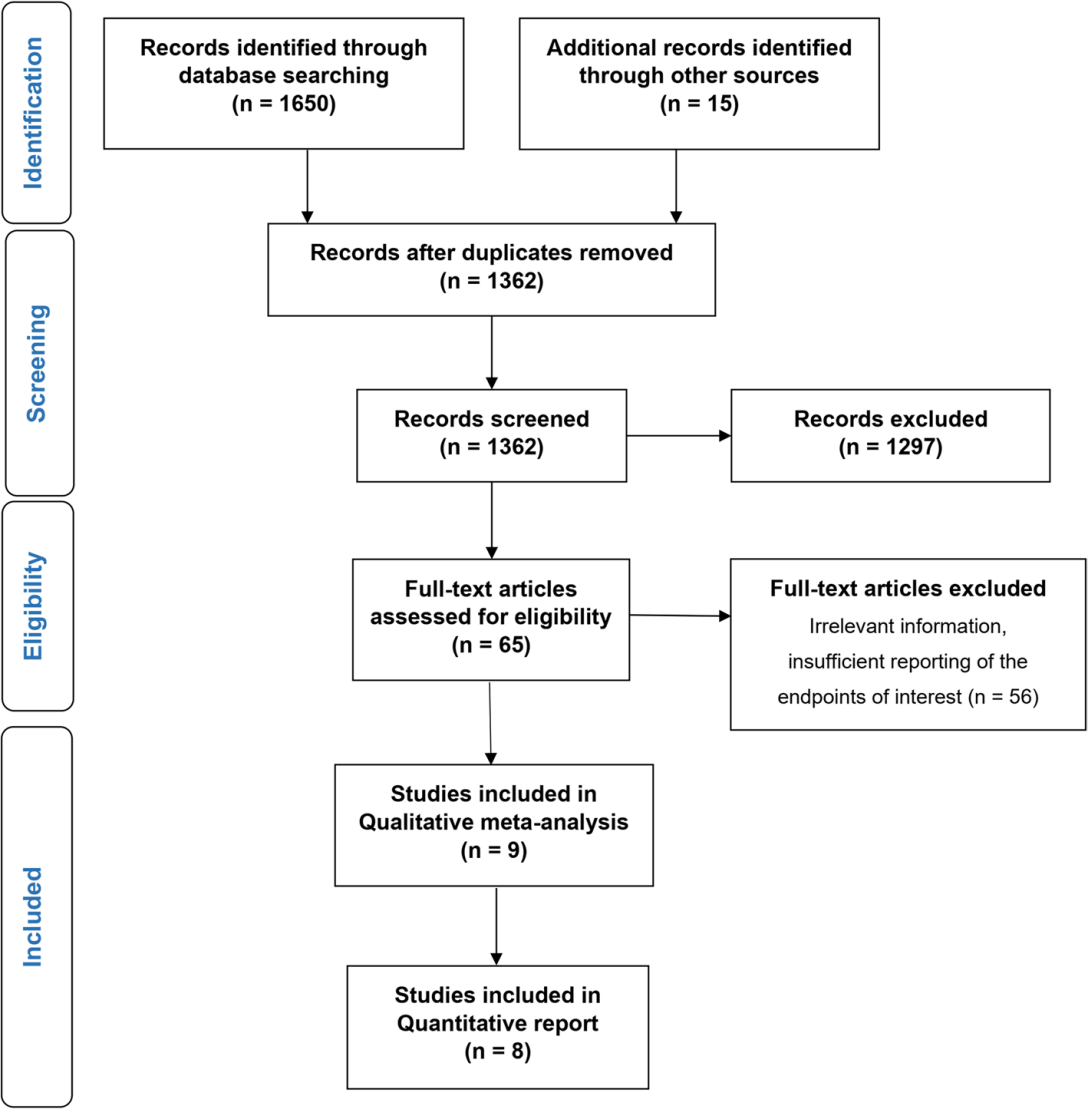
Flow chart of study

The literature search retrieved 1362 studies after duplicates were excluded. Evaluation of titles and abstracts excluded a further 1297 articles. Of the 65 full-text articles assessed for eligibility, 56 were excluded for various reasons, including insufficient data on survival. Finally, nine articles were included in the qualitative and eight articles in the quantitative meta-analysis (Fig. 1). All included studies were retrospective and reported on 44,219 patients (2243 in the statin group and 41,976 in the non-statin group). All studies were published between 2012 and 2021. The certainty of evidence for the outcomes, assessed using the GRADE approach, was moderate.

### Qualitative report

Of the nine included studies **(**Table 1**),** only Yang and Young studies [26, 27] classified their patient based on Barcelona clinic liver cancer (BCLC) Staging. In the young study, of the 430 participants, approximately 59% were in stage A, 36 in stage B, and the rest in stage C. Only patients with stage 0 or A were included in Yang study. Four studies [20, 23, 26, 28] had sufficient information regarding recurrence free survival in statin and no-statin groups. According to available data in included studies, we performed two separate meta-analyses of HRs and ORs. Wu LL study [22] was excluded from meta-analyses of HRs, because statin use was not reported separately in patients who underwent surgery.

**Table 1:** Included studies in qualitative analysis

Author	Year	Country	Type	Statin group	Non-statin group	LRx or LTx	Remarks and main findings
Yang [26]	2021	Taiwan	Retrospective	46	774	LRx	- Statins included atorvastatin, fluvastatin, pitavastatin, and rosuvastatin. - Statin use significantly reduced HCC recurrence (HR: 0.354; *p* value < 0.001). - The statin group had higher RFS than the non-statin group after propensity score matching.
Young [27]	2020	Taiwan	Retrospective	30	400	LRx	- Statins included lovastatin, fluvastatin, rosuvastatin, atorvastatin, and pravastatin. - Statin use (HR = 0.50; 95% CI = 0.27–0.94, *p* = 0.031) was significantly associated with decreased recurrence in univariate analysis.
Cho [23]	2019	Korea	Retrospective	112	235	LTx	- The types of statins used in the study are not specified. - Statin therapy was associated with a reduced risk of HCC recurrence (OR = 0.38, 95% C I = 0.16–0.91).
Nishio [28]	2018	Japan	Retrospective	43	600	LRx	- 17 patients used pravastatin, 15 patients atorvastatin, 7 patients rosuvastatin, and 4 patients pitavastatin. - Significant improvement of both RFS (5-year RFS, 55.4% in the statin group versus 25.0% in the non-statin group) and OS (5-year OS, 73.1% versus 56.7%, respectively) in perioperative statin users.
Kawaguchi [20]	2017	Japan	Retrospective	31	703	LRx	- Statins included pravastatin, simvastatin, fluvastatin, pitavastatin, atorvastatin, and rosuvastain. - The RFS was significantly higher in the statin than non-statin group (*P* < 0.001): the 1-, 3-, and 5- year RFS were 87.1, 76.7, and 76.7%, respectively, in the statin group, and 65.3, 40.6, and 32.9%, respectively, in the non-statin group. - The OS was not significantly different between the groups.
Wu LL [22]	2016	Taiwan	Retrospective	934	17,958	LRx, and other treatments	- The types of statins used in the study are not specified. - Better OS with surgery and statin use compared with RFA/PEI and statin use (*p* = 0.0003 and *p* = 0.019, respectively, for stages I and III).
Lee [29]	2016	Taiwan	Retrospective	132	2078	LRx	- The types of statins used in the study are not specified. - Use of statins and NSAIDs also can reduce the risk of recurrence of HCC and mortality after surgery.
Yeh [30]	2015	Taiwan	Retrospective	740	14,834	LRx	- The types of statins used in the study are not specified. - The use of statin can significantly reduce risk of recurrent HCC (HR, 0.51; 95% CI, 0.42–0.61; *P* < 0.001).
Wu Cy [31]	2012	Taiwan	Retrospective	175	4394	LRx	- The use of statin was significantly associated with lower risk of tumor recurrence (HR: 0.68; 95% CI: 0.53–0.87; *p* = 0.002). - The types of statins used in the study are not specified.

*Abbreviations*: *LRx* liver resection, *LTx* liver tranplantation, *HR* Hazard ratio, *OR* Odds ratio, *RFS* recurrence-free survival, *OS* overall survival, *RFA* radiofrequency ablation, *PEI* percutaneous ethanol injection

The quality assessment of included studies is shown in Table 2**.** All included studies had an overall MINORS score of less than 20, indicating a considerable risk of bias. All included studies had a high risk of biased assessment of the study endpoint because they were non-randomized and non-blinded. In addition, all included studies were retrospective so did not prospectively calculate the sample size.

**Table 2:** Assessment of the quality of studies included in qualitative and quantitative analyses

***Authors***	***Q1***	***Q2***	***Q3***	***Q4***	***Q5***	***Q6***	***Q7***	***Q8***	***Q9***	***Q10***	***Q11***	***Q12***	***Score*** ^a^
Yang [26]	2	1	0	2	2	2	2	0	2	0	2	2	17
Young [27]	2	2	0	2	1	2	2	0	2	0	2	2	17
Cho [23]	2	2	0	2	1	2	2	0	2	0	2	2	17
Nishio [28]	2	1	0	2	1	2	2	0	2	0	2	2	16
Kawaguchi [20]	2	2	2	2	1	2	2	0	2	0	2	2	19
Wu LL [22]	2	1	1	2	1	2	1	0	2	0	2	2	16
Lee [29]	2	1	0	2	1	2	1	0	2	0	2	2	15
Yeh [30]	2	1	0	2	1	2	2	0	2	0	1	2	15
Wu Cy [31]	2	1	1	2	1	2	1	0	2	0	2	2	16

Q1. A clearly stated aim Q2. Inclusion of consecutive patients Q3. Prospective collection of data Q4. Endpoints appropriate to the aim of the study Q5. Unbiased assessment of the study endpoint Q6. Follow-up period appropriate to the aim of the study Q7. Loss to follow up less than 5% Q8. Prospective calculation of the study size Q9. An adequate control group Q10. Contemporary groups Q11. Baseline equivalence of groups Q12. Adequate statistical analyses

^a^ The items are scored 0 (not reported), 1 (reported but inadequate) or 2 (reported and adequate). The best total score is 16 for non-comparative studies and 24 for comparative studies

### Quantitative analysis

**Fig. 2:**
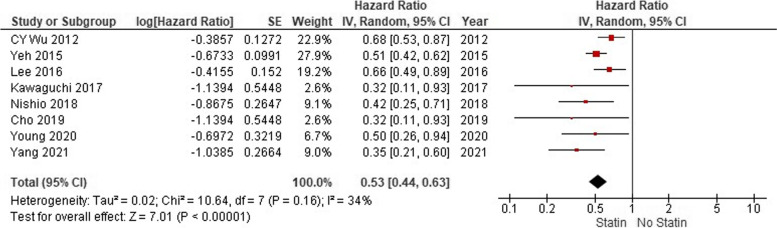
Pooled analysis of hazard ratios for recurrence of HCC

In 8 studies with totally 25,327 participants, association between statin use and HCC recurrence was reported as HRs. After pooling of HRs of HCC recurrence using a random-effect model, the rate of recurrence was significantly lower in patients who received statins before their surgery (HR: 0.53; 95% CI: 0.44–0.63; *p* < 0.001; Fig. 2). No significant heterogeneity was seen between the studies in this regard (I^2^ = 34%; *P* = 0.16).

**Fig. 3:**
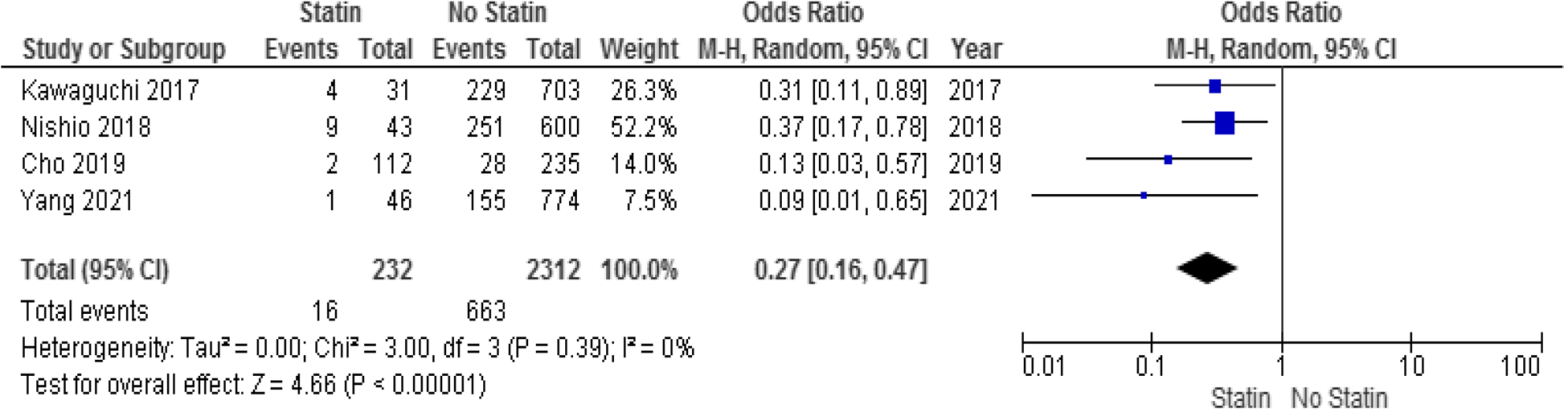
Forest plot for HCC recurrence 1 year after liver surgery

HCC recurrence after surgery was reported in 2544 patients from four studies (232 patients in the statin group and 2312 patients in the non-statin group). HCC recurrence 1 year after surgery occurred in 16 patients (6.89%) in the statin group and in 663 patients (28.67%) in the non-statin group. Meta-analysis indicated that statin use decreased HCC recurrence 1 year after surgery (OR: 0.27; 95% CI: 0.16–0.47; *p* < 0.001; Fig. 3) using a random-effects model. There was no considerable heterogeneity among the studies (I^2^ = 0%; *p* = 0.39).

**Fig. 4:**
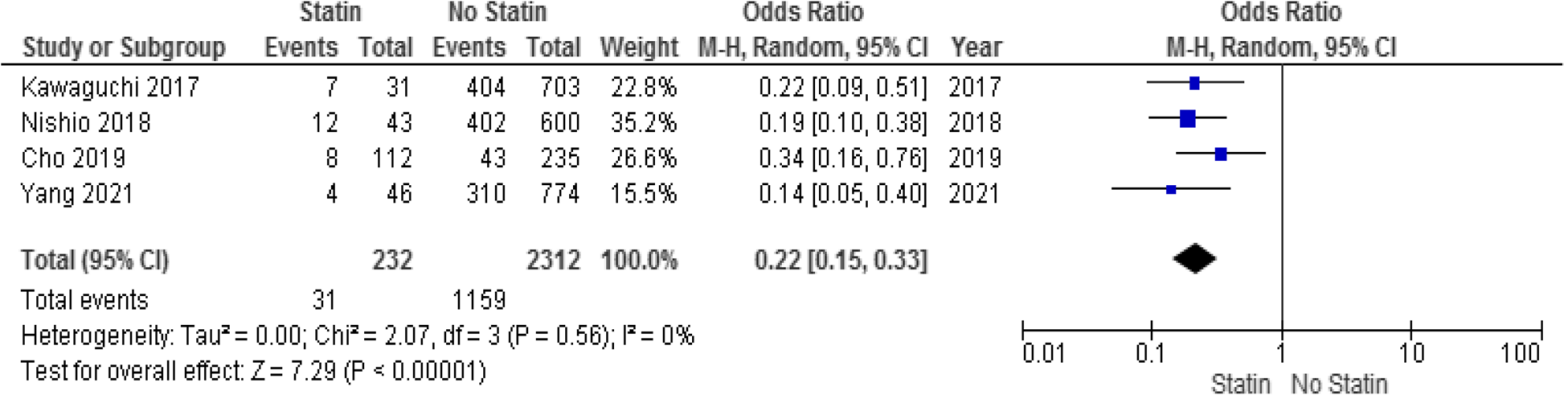
Forest plot for HCC recurrence 3 years after liver surgery

HCC recurrence 3 years after surgery was reported in 31 patients (13.3%) in the statin group and in 1159 patients (50.1%) in the non-statin group. Meta-analysis indicated that statin use decreased HCC recurrence 3 years after surgery (OR: 0.22; 95% CI: 0.15–0.33; *p* < 0.001; Fig. 4) using a random-effects model. There was no considerable heterogeneity among the studies (I^2^ = 0%; *p* = 0.56).

**Fig. 5:**
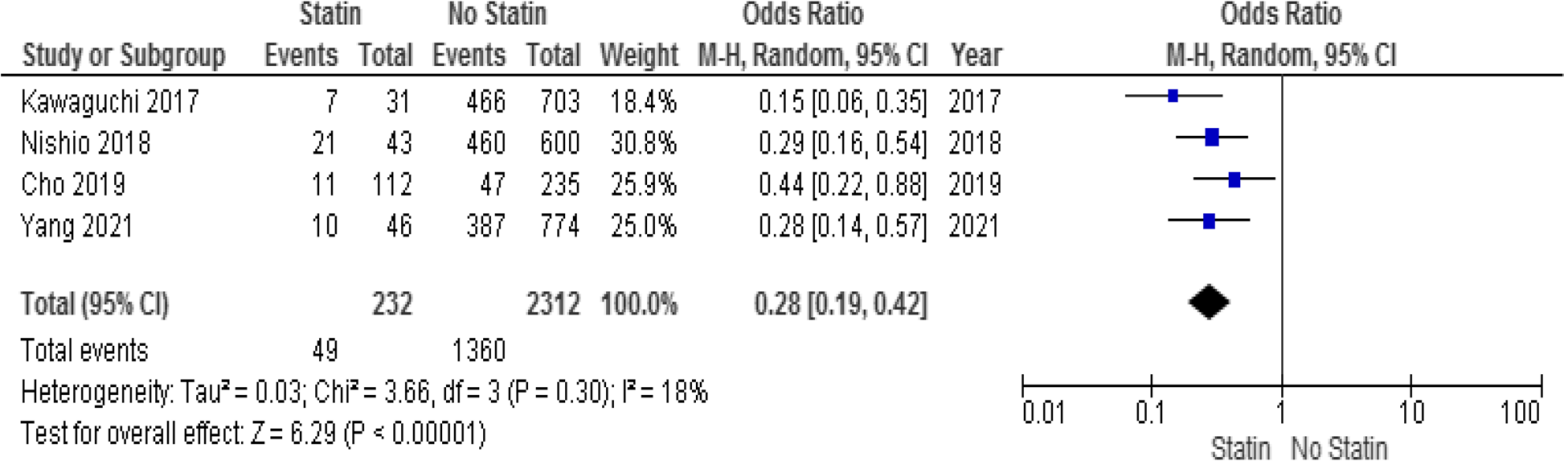
Forest plot for HCC recurrence 5 years after liver surgery

HCC recurrence 5 years after surgery was reported in 49 patients (20.9%) in the statin group and in 1360 patients (58.8%) in the non-statin group. Meta-analysis indicated that statin use decreased HCC recurrence 5 years after surgery (OR: 0.28; 95% CI: 0.19–0.42; *p* < 0.001; Fig. 5) using a random-effects model. There was no considerable heterogeneity among the studies (I^2^ = 18%; *p* = 0.30).

## Discussion

HCC comprises more than 80% of primary liver cancers [32] and is the third most deadly cancer worldwide. In Europe, it is the seventh leading cause of death [33, 34]. In the recent years, the etiology and characteristics of HCC patients is widely changed. New radiological methods in the diagnosis of HCC patients and differentiating primary and recurrent nodules have been introduced [35]. In addition, proper surveillance programs are performed around the world that help to identify patients in the lower stages of the disease and enable early treatment of the patients [36]. The OS of patients is higher if the diagnosis is made early [9], but high HCC recurrence rates after surgical treatment still remain a big challenge [12, 37]. New methods are urgently needed to reduce recurrence, thereby improving long-term surgical outcomes and reducing healthcare-related costs in the future.

Because of the virulent character of HCC, prevention plays a significant role in treatment [38]. Different studies have investigated the protective effect of several drugs against HCC recurrence, including statins, aspirin, and anti-diabetic agents [8]. Statins are classified into lipophilic and hydrophilic statins and have been used extensively to prevent and treat cardiovascular diseases [39, 40]. Studies have recently demonstrated that statins can reduce the risk of many cancers, including liver cancers [39, 41]. Furthermore, statins can inhibit progression of liver fibrosis and cirrhosis in HCC patients [42] and can reduce the risk of HCC in patients with Hepatitis C Virus (HCV) infection and also with nonalcoholic fatty liver disease (NAFLD) [43, 44]. Lipophilic statins seem to be more effective at preventing HCC than hydrophilic statins are [39]. Fluvastatin has been revealed as the most effective drug in reducing HCC risk [8].

In this systematic review and meta-analysis, we investigated whether statins can reduce HCC recurrence following liver surgery. We showed that the recurrence was lower at one, three, and 5 years after surgery in patients with HCC who underwent liver surgery in combination with statin treatment than in patients who underwent liver surgery without statin treatment. These results indicate that statins should be considered effective at reducing the recurrence of HCC tumors.

Statins may reduce the risk of cancer via several mechanisms, including inhibiting oncogenic pathways, promoting tumor-specific apoptosis, inhibiting the proteasome pathway, inhibiting hepatitis virus replication, and reducing cholesterol synthesis [45, 46]. Statins can also decrease endothelial dysfunction, intrahepatic vasoconstriction, inflammation, and fibrosis [47–49]. The anti-inflammatory and immunomodulatory effects of statins allow them to inhibit harmful inflammatory and immunologic responses that may promote cancer [47–49]. The effect of statins on liver regeneration and ischemia-reperfusion injury after extensive hepatectomy has been investigated in animal models. These studies showed that these drugs can improve outcomes by facilitating regeneration and by inhibiting the harmful inflammatory response [50, 51]. In a pilot clinical study, preoperative oral atorvastatin therapy for 3 days prior to liver resection reduced the harmful immunologic and inflammatory responses due to ischemia-reperfusion injury [52].

Patients who are not eligible for liver resection can be treated with more conservative options [22]. Wu et al. [22] reported that combining statin treatment with these conservative methods improves the survival of patients with advanced HCC. In patients with a contraindication for these conservative therapies, especially those with HBV/HCV, palliative treatment with statins can reduce the mortality rate [22]. It seems that combining statins and conservative therapies in patients who cannot undergo surgical therapies can improve the HCC prognosis [22, 53]. According to Aaron et al., statins can improve the survival of HCC patients when administered both before or after HCC is diagnosed [54].

HCC recurrence is detected in half of patients 3 years after liver surgery [55]. Recurrent disease is not easily treatable, so it is important to prevent recurrence after resection [20]. Studies have shown that statins can reduce HCC recurrence rate by reducing viremia in patients with HBV and HCV. Possible mechanisms include reducing pro-inflammatory cytokines in serum [8, 56, 57], reducing the virulent potency of viral infections [20, 57], or inhibiting cirrhotic progression [57]. However, the type of antiviral regimen can also affect the recurrence risk and survival of HCV-related HCC and need to be evaluated in further studied on statins [58].

Preventing HCC recurrence after liver transplantation is also an important issue [59] but has not been well investigated. Statins have several side-effects (including myalgia and myotoxicity that may lead to rhabdomyolysis), especially when administered at high doses. These side-effects need to be properly investigated in post-transplant patients [60, 61]. The prevalence of post-liver transplant dyslipidemia is 16–66% worldwide [47]. Statins decrease lipidemia in patients after liver transplantation, thereby preventing cardiovascular events [60].

There are some limitations to the present systematic review and meta-analysis. First, all of included studies are from Asia-pacific area and it have been mentioned as a limitation of the present study, that can affect the results of this study. Moreover, we found no RCT that compared the clinical outcomes of liver surgery between statin and non-statin groups. Furthermore, the timing, dosage, and type of statin is important to evaluate the outcomes; however, this information was not provided in every study. Further well-designed, large-scale RCTs are needed to determine whether statin therapy prevents HCC recurrence after liver resection or transplantation.

In conclusion, statins increased disease-free survival of patients with HCC after liver surgery. They may reduce HCC recurrence after liver surgery by chemoprevention effects. Unfortunately, the existing evidence is still too limited (small study populations, retrospective study designs, and single-center studies) to confirm a role for statins in reducing disease recurrence in HCC patients. Further randomized clinical trials should confirm the effectiveness of statins in preventing HCC recurrence after liver surgery, and should determine the importance of different types of surgery and types of statins.

## Data Availability

Only publicly available data were used in this study, and data sources and handling of these. data are described in the Materials and Methods and in the additional files. Further information is available from the corresponding author upon request.

## Abbreviations

BCLC: Barcelona clinic liver cancer
GRADE: Grades of Research, Assessment, Development, and Evaluation
HCC: Hepatocellular Carcinoma
HCV: Hepatitis C Virus
HMG-CoA: Hydroxy-Methyl Glutaryl Coenzyme A
HR: Hazard Ratio
MINORS: Methodological Index for Non-Randomized Studies
NAFLD: Nonalcoholic Fatty Liver Disease.
OR: Odds Ratio
PRISMA: Preferred Reporting Items for Systematic Reviews and Meta-Analyses
